# Small extracellular vesicles derived from embryonic stem cells restore ovarian function of premature ovarian failure through PI3K/AKT signaling pathway

**DOI:** 10.1186/s13287-019-1508-2

**Published:** 2020-01-03

**Authors:** Mengyu Liu, Yu Qiu, Zhuowei Xue, Ruoyu Wu, Jie Li, Xin Niu, Ji Yuan, Yang Wang, Qingkai Wu

**Affiliations:** 1 Department of Obstetrics and Gynecology, Shanghai Jiao Tong University Affiliated Sixth People’s Hospital, No.600 Yishan Road, Shanghai, 200233 China; 2 Medical College of Soochow University, Suzhou, 215006 China; 3 Institute of Microsurgery on Extremities, Shanghai Jiao Tong University Affiliated Sixth People’s Hospital, No.600 Yishan Road, Shanghai, 200233 China

**Keywords:** Small extracellular vesicle derived from embryonic stem cells (ESCs-sEVs), Premature ovarian failure (POF), PI3K/AKT signaling pathway, Ovarian function

## Abstract

**Background:**

Premature ovarian failure (POF) has a great impact on reproductive endocrine function in females, and it is an important cause of infertility. Previous studies have demonstrated that small extracellular vesicles (sEVs) derived from stem cells play an important role in tissue regeneration. This study aimed to investigate the therapeutic effect of sEVs derived from embryonic stem cells (ESCs-sEVs) on damaged ovaries and explore the underlying molecular mechanisms.

**Methods:**

Mice POF models were established by injecting mice with cyclophosphamide and busulfan. Then, ESCs-sEVs were intravenously transplanted into POF mice. The plasma of mice was harvested at 1 and 2 weeks after treatment to analyze the levels of anti-Mullerian hormone (AMH), estradiol (E_2_), and follicle stimulating hormone (FSH) by ELISA. The morphology of ovaries and follicles was observed by H&E staining, and apoptosis of granulosa cells was detected by TUNEL. In vitro, EdU and CCK-8 tests were used to evaluate the proliferation of cultured granulosa cells stimulated by ESCs-sEVs. Western blotting was used to determine the expression of PI3K/AKT and apoptotic-related proteins.

**Results:**

After transplantation of ESCs-sEVs, the levels of serum sex hormones recovered to normal levels. In addition, the number of follicles was significantly increased, and the number of apoptotic cells was decreased. The results in vitro revealed that ESCs-sEVs could significantly improve the proliferation rate of granulosa cells and increase the expression of phosphorylated PI3K and AKT. Meanwhile, the positive effect on proliferation and the negative effect on apoptosis observed in granulosa cells were obviously decreased when the PI3K/AKT signaling pathway was inhibited.

**Conclusion:**

Our findings suggested that ESCs-sEVs could improve ovarian function by regulating the PI3K/AKT signaling pathway, which could provide a promising clinical therapy for POF.

## Introduction

Premature ovarian failure (POF) is a gynecological disease that is associated with a myriad of complex etiologies, and it has a serious impact on women’s fertility and quality of life. The incidence of POF is increasing each year and tends to increase with advancing age [1]. Currently, hormone replacement therapy (HRT) is clinically available to relieve menopausal symptoms. However, the long-term application of HRT is limited due to its associated side effects,thus, it is imperative to find new treatment strategies.

In recent years, with the development of regenerative medicine, mesenchymal stem cells (MSCs) have been reported to play a vital therapeutic role in the treatment of POF [2–4]. However, there could be a risk of immune rejection during allogeneic MSC transplantation. Additionally, autotransplantation of MSCs faces the problems of an invasive operation and insufficient sources of aged tissue. Embryonic stem cells (ESCs) have the unique ability to proliferate indefinitely and are pluripotent, making them a promising therapeutic candidate in aging-related disease [5].

Extracellular vesicles (EVs) are secreted by a variety of cells that participate in cell-to-cell communication and regulate receptor cell function by transferring small molecular proteins, miRNAs, lncRNAs, and cytokines [6]. Small EVs are defined as EVs with diameters that are less than 200 nm [7]. Accumulating evidence indicates that EVs hold a similar function to their parental cells in promoting tissue regeneration [8]. Meanwhile, they can avoid potential risks during the cell transplantation process, providing a safer substitution for therapeutic application in the clinic [9, 10]. Our previous study revealed that ESCs-sEVs accelerate the healing process of aged mice by promoting local angiogenesis and reversing the senescence of endothelial cells [11]. In addition, the ESCs-sEVs play an important part in the treatment of osteoarthritis [12]. In addition, the unlimited proliferation and self-renewal characteristics of ESCs ensure that ESCs-sEVs can be collected in large numbers and can be commercially produced. Currently, the effect of ESCs-sEVs on POF has not yet been investigated. Hence, we speculate that ESCs-sEVs could play a protective role in POF.

In our study, we first observed that ESCs-sEVs could significantly promote recovery of ovarian function, as evidenced by the remarkably increased number of growth follicles and the restoration of hormone levels. Further in vitro experiments confirmed that ESCs-sEVs could inhibit granulosa cell apoptosis. Further exploration of the underlying mechanism revealed that ESCs-sEVs function in POF through activation of the PI3K/AKT signaling pathway.

## Methods

### Isolation and identification of ESCs

Human ESCs (H9) were purchased from the Institute of Biochemistry and Cell Biology of Chinese Academy of Sciences and were cultured under standard conditions with mTeSR1 medium (StemCell Technologies). The markers of ESCs (Nanog, Oct4, SSEA4, and TRA-1-81) were evaluated by immunostaining.

### Isolation and identification of ESCs-sEVs

The cell culture supernatant of ESCs (without fetal bovine serum (FBS)) was collected, and ESCs-sEVs were isolated by differential centrifugation and ultracentrifugation as described previously. Briefly, the medium was centrifuged at 300*g* for 15 min and 2000*g* for 30 min to remove dead cells. The medium was then ultracentrifuged twice at 100,000*g* for 2 h to remove the supernatant, and finally, the obtained pellet was resuspended in PBS.

The size distribution and concentration of ESCs-sEVs were measured by a Flow Nano Analyzer. Next, the morphology of ESCs-sEVs was observed by transmission electron microscopy (TEM, Hitachi H-7650). The markers of ESCs-sEVs, CD9 (1:1000; Epitomics), CD63 (1:1000, Epitomics), and TSG-101 (1:1000, Abcam), were detected by Western blotting. In addition, the expression of cis-Golgi matrix protein GM130 (1:1000, Abcam), Actin (1:5000, Abcam), and Lamin A/C (1:1000, Servicebio) were assessed in ESCs-sEVs and ESCs to detect the purity of ESCs-sEVs.

### Isolation and culture of granulosa cells

Eight-week-old female C57BL/6 mice (*n* = 5) were purchased from the Experimental Animal Centre of Shanghai Six People’s Hospital Affiliated with Jiaotong University. All mice were sacrificed by cervical dislocation, which was followed by isolation of ovaries from mice under sterile conditions; ovaries were then washed with sterile phosphate-buffered saline (PBS, HyClone, USA) three times and were cut into pieces. The tissue was digested with 1 mL 0.1% hyaluronidase (Sigma, USA) at 37 °C for 5 min, and then, 4 mL PBS was added to terminate the digestion. After that, the suspension was filtered by a 200-mesh cell strainer and was followed by two washes with PBS. Thereafter, the supernatant was discarded, and the pellet was resuspended in Dulbecco’s modified Eagle’s medium that contained Ham’s F12 Medium (DMEM: F12) (1:1) with 10% (v/v) FBS (Gibco, USA) and 1% penicillin and streptomycin. The suspension was seeded in 6-well plates and incubated at 37 °C in 5% CO_2_. The media was changed, and the cells were washed with PBS to discard the non-adherent cells after 24 h.

The passage 2 ovarian granulosa cells were seeded at a density of 1 × 10^5^ cells/well into a six-well plate. After 24 h, the culture medium was discarded and treated with DMEM/F12 only for 24 h to starve the cells. The cells were then divided into four groups. In the ESCs-sEVs group, ESCs-sEVs were added to the culture medium at a density of 1 × 10^8^ particles/mL. LY294002 was used as an AKT phosphorylation inhibitor. In the LY294002 group, the inhibitor was dissolved in DMSO and added into the culture medium at a concentration of 50 μM, as described in previous studies [13, 14]. In the ESCs-sEVs + LY294002 group, both ESCs-sEVs and LY294002 were added to the culture medium at the same concentration as mentioned above. In the control group, the granulosa cells were cultured with only DMEM/F12 medium.

### POF mouse model and injection of ESCs-sEVs

Eight-week-old and naturally aged (14 months old) female C57BL/6 mice were obtained, and all procedures were approved by the Experimental Animal Centre of Shanghai Six People’s Hospital Affiliated with Jiao Tong University. The young mice were divided into 3 equal groups: the POF (*n* = 25) and ESCs-sEVs (*n* = 25) group received a single treatment of cyclophosphamide (CTX, Sigma, 120 mg/kg) and busulfan (BUS, Sigma, 30 mg/kg); CTX and BUS were dissolved in 0.2 mL DMSO and were administered by intraperitoneal injection to establish the POF model as described previously [15]. The control group (*n* = 25) received a single intraperitoneal injection with an equal amount of saline. The success of the POF model was confirmed by continuously observing mouse vaginal smears and mouse body weight for 14 days after injection. After that, the mice in the ESCs-sEVs group were injected via the tail vein intravenously with 1 × 10^8^/mL ESCs-sEVs in a volume of 0.2 mL PBS three times once every 2 days. Similar to the ESCs-sEVs group, the POF and the control group were injected with 0.2 mL PBS intravenously three times. The mice were sacrificed on the day before injection (0 week) and at 1 and 2 weeks after the last injection, and the peripheral blood serum and ovaries were harvested.

The natural aged mice were divided into two groups, in which the aging + ESCs-sEVs group received the same injection as the ESCs-sEVs group, and the aging-control group received the same injection as that of the previously mentioned control group. The mice were sacrificed 2 weeks after the last injection, and the peripheral blood serum and ovaries were harvested.

### ELISA analysis of E2, FSH, and AMH

ELISA was performed according to the manufacturer’s instructions for anti-Mullerian hormone (AMH), estradiol (E_2_), and follicle stimulating hormone (FSH). Briefly, 50 μL of each mouse serum was added to each well of a coated 96-well plate and then incubated for 60 min at 37 °C. After the reaction was finished, the wells were washed with wash buffer three times, which was followed by the addition of HRP-conjugated antibodies into each well and 60 min of incubation at 37 °C. Finally, the stop buffer was added, and the optical density (OD) was measured at a wavelength of 450 nm. After that, 5 independent samples from each group were placed in 3 wells for replicate experiments.

The suspension of granulosa cells from each group was collected separately after 24 h, 48 h, and 72 h by centrifugation at 1500 r/min for 5 min, which enabled the detection of AMH levels using ELISA kits. Meanwhile, the granulosa cell culture medium of each group was taken as a control. The OD value was measured by a spectrophotometer (iMarkTM; Bio-Rad, USA) and analyzed. Three replicates were obtained for each group.

### Ovarian follicle counts and morphological analysis

The mice were sacrificed at 0, 1, and 2 weeks after treatment. Their ovaries were collected and fixed in 4% paraformaldehyde for at least 24 h. After dehydration and paraffin embedding, the ovaries were cut to produce 5 μm sections. Three representative sections of each ovary were selected. The sections were stained with hematoxylin-eosin and the primordial, primary, secondary, antral and atretic follicles were counted under light microscopy.

### TUNEL analysis

The frozen sections of mouse ovaries were processed using a Cell Death Detection Kit (Beyotime, China). Briefly, the sections were fixed with 4% paraformaldehyde for 30 min and then were permeabilized with 0.3% Triton-X-100 for 5 min. Next, TUNEL detection solution was added, and the sections were incubated at room temperature without light. The nucleus was labeled by incubating sections with Hoechst 33324 for 5 min. Finally, the sections were analyzed using fluorescence microscopy (DMI 6000, Leica Micro systems, Buffalo Grove, IL, USA). The apoptotic index was defined as the percentage of TUNEL-positive areas per slide for quantitative analysis.

### Cell proliferation of granulosa cells

Passage 2 granulosa cells were seeded into a 96-well plate at 4000 cells/well. After 24 h, the culture medium was changed as mentioned above. Then, 10 μL of a Cell Counting Kit-8 (CCK-8, Beyotime) solution was added into each well according to the manufacturer’s instructions. The plates were incubated at 37 °C in 5% CO_2_ for 2 h. The absorbance was measured at 450 nm by a microplate reader and was then analyzed. The same procedure was performed on days 1, 2, 3, 4, and 5 to analyze the proliferation of granulosa cells.

Passage 2 granulosa cells were seeded into 6-well plates at a density of 1 × 10^4^ cells/well. When the cells reached 70% confluence, the culture medium was changed as described above and removed after 72 h. Next, an EdU-Click 488 proliferation kit (Sigma-Aldrich, USA) was used to analyze cells according to the manufacturer’s instructions. The cells were observed and imaged under a fluorescence microscope (DMI 6000, Leica Micro systems, Buffalo Grove, IL, USA).

### Immunofluorescence staining

Cells were cultured on a glass slide and fixed in 4% paraformaldehyde for 15 min. The cells were permeabilized with 0.1% Triton X-100 for 10 min, and non-specific antibody interaction sites were then blocked for 15 min. Next, the cells were incubated at 4 °C overnight with primary antibodies, Nanog (ab109250, Abcam, USA), Oct4 (ab200834, Abcam, USA), SSEA4 (ab16287, Abcam, USA), and TRA-1-81 (ab16288, Abcam, USA), to identify ESCs and follicle stimulating hormone receptor (FSHR, 22665-1-AP, Proteintech, China) to identify granulosa cells. On day 2, the cells were incubated with a secondary antibody in a darkroom for 1 h. Finally, the cell nuclei were labeled with Hoechst 33324 for 1 min. After that, the slides were observed and imaged using a fluorescence microscope (DMI 6000, Leica Micro systems, Buffalo Grove, IL, USA).

### Western blotting analysis

The protein was extracted from granulosa cells using RIPA lysis buffer (Beyotime, China) with proteinase and phosphatase inhibitors. The protein concentration was measured by a BCA protein assay kit (Beyotime, China). The samples were denatured at 95 °C for 10 min with 5× protein-loading buffer. The protein was separated by 10% SDS-PAGE and then was transferred onto 0.22-μm PVDF membranes (Millipore, USA). Next, the membranes were blocked with 5% skim milk for 1 h and were then incubated with primary antibodies at 4 °C overnight. Subsequently, the membranes were washed three times and incubated with HRP-conjugated goat anti-rabbit or goat anti-mouse secondary antibodies (1:3000, Abcam, USA) for 1 h. Finally, the proteins were detected by enhanced chemiluminescence and were imaged by an Image Quant LAS 4000 mini biomolecular imager (Bio-Rad, USA).

### Statistical analysis

Each experiment in this study was performed at least three times. All results are presented as the mean ± SD and analyzed using SPSS 22.0 software. One-way analysis of variance (ANOVA) and Student’s *t* tests were used for statistical comparisons among different groups, and *P* < 0.05 was considered to be significantly different.

## Results

### Characterization of ESCs and ESCs-sEVs

**Fig. 1:**
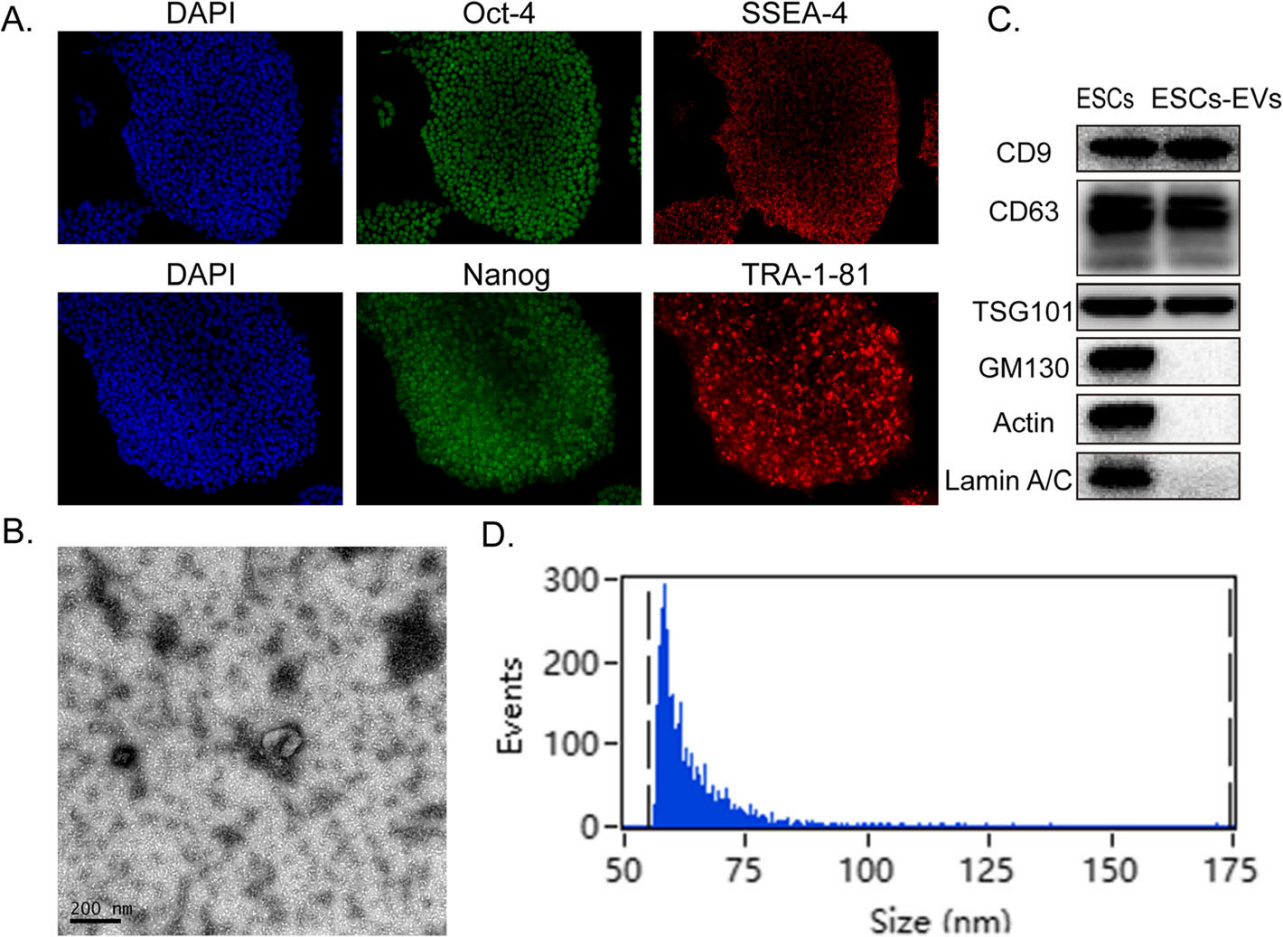
Characterization of ESCs and ESCs-sEVs. **a** Immunofluorescence detected the pluripotency markers in ESCs, including Oct-4, SSEA-4, Nanog, and TRA-1-81. Scale bars = 50 μm. **b** The morphology of ESCs-sEVs by TEM. Scale bars = 200 μm. **c** ESCs-sEVs were positive for CD9, CD63, and TSG101 and negative for GM130, Actin, and Lamin A/C, as shown by Western-blotting analysis. **d** Particle size distribution of ESCs-sEVs was determined by Flow Nano Analyzer

Immunostaining analysis revealed the expression of ESCs with specific pluripotency-related markers, including Oct4, SSEA4, Nanog, and TRA-1-81 (Fig. 1a). ESCs-sEVs were extracted from the supernatant of ESCs culture medium. The morphology of cup-shaped vesicles was observed under TEM (Fig. 1b). Western blotting demonstrated that the ESCs-sEVs were positive for CD9, CD63, and TSG101, and they were negative for Golgi membrane bound protein GM130, Actin, and Lamin A/C (Fig. 1c). Flow Nano Analyzer showed the size distribution of ESCs-sEVs to range from approximately 50 to 75 nm at a concentration of 2.6 × 10^9^ particles/mL (Fig. 1d). All the characterization of ESCs and ESCs-sEVs meets the criteria for defining them as such [11].

### ESCs-sEVs restored ovarian function in a POF mouse model

**Fig. 2:**
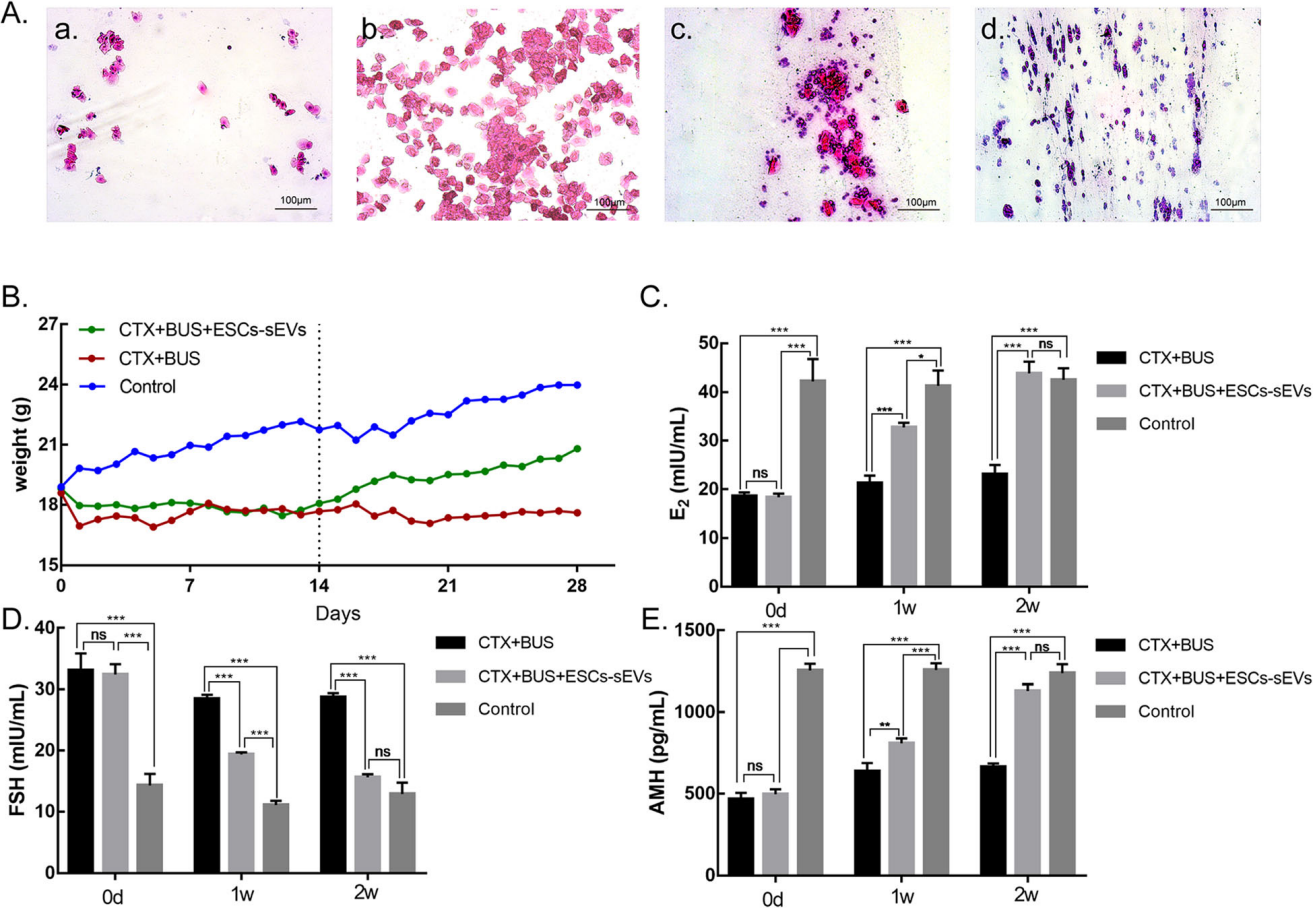
ESCs-sEVs contributed to the estrous cycle, body weight, and hormone levels in mice. **A** Estrous cycle of mice: (a) proestrus, (b) estrus, (c) metestrus, and (d) diestrus. **B** The weight of mice with ESCs-sEVs gradually increased to normal levels, while the weight of the CTX + BUS group gradually decreased to stable levels. The dashed line indicates mice that received treatment for 14 days. **C** E_2_ was significantly increased compared to the CTX + BUS group. **D** FSH was significantly decreased compared to the CTX + BUS group. **E** AMH was significantly increased compared to the CTX + BUS group. Scale bars = 100 μm. (Data are presented as the mean ± SD, *P < 0.05, **P < 0.01, ***P < 0.001; ns, no significance)

To confirm the successful establishment of the model, the body weight and vaginal smear of each group of mice were assessed every morning at 8 am. The regular estrous cycle was approximately 4–6 days in the control group: the proestrus was 17–24 h, estrus was 9–15 h, metestrus was 10–14 h, and diestrus was 60–70 h (Fig. 2A (a–d)). The estrous cycle was disturbed after establishing the POF model, in which most of the mice stayed in the estrous phase and lost the periodic change (including stagnation and prolongation). The results suggest that the POF model was successfully established. In the ESCs-sEVs group, the estrous cycle was gradually restored to normal after treatment, while the mice in the CTX + BUS group still exhibited disordered estrous cycle. The body weight was not significantly different among the 3 groups before intraperitoneal injection of CTX + BUS. At 1–14 days after establishment of the POF model, the mice in the ESCs-sEVs and CTX + BUS groups had both gradually lost weight compared to the mice in the control group. After ESCs-sEVs treatment, the weight of the ESCs-sEVs group showed a gradual increase to nearly normal levels, while the mice in the CTX + BUS group continued to lose weight until reaching a stable level (Fig. 2B).

Next, to evaluate the effect of ESCs-sEVs on the sex hormones of POF mice, the hormone levels of E_2_, FSH, and AMH in each group were tested by ELISA at different time points (Fig. 2C–E). More detailed data are shown in Additional file 1. On day 14 after the establishment of POF model by CTX + BUS (the day before treatment), the E_2_ and AMH levels were much lower in both the CTX + BUS and ESCs-sEVs groups than they were in the control (E_2_—18.56 ± 0.78 mIU/mL vs 42.23 ± 4.53 mIU/mL, and 18.35 ± 0.75 mIU/mL vs 42.23 ± 4.53 mIU/mL, respectively, *P* < 0.001; AMH—466.88 ± 38.98 pg/mL vs 1254.13 ± 41.67 pg/mL, and 496.28 ± 30.58 pg/mL vs 1254.13 ± 41.67 pg/mL, respectively, *P* < 0.001). Conversely, the FSH level was significantly higher than it was in the control group (33.08 ± 27.68 mIU/mL vs 14.30 ± 1.89 mIU/mL, and 32.42 ± 1.66 mIU/mL vs 14.30 ± 1.89 mIU/mL, respectively, *P* < 0.001), suggesting the successful establishment of a POF animal model. Similar results could be observed in the ovaries with H&E staining. At 1 and 2 weeks after administration of ESCs-sEVs, the levels of E_2_ and AMH in the ESCs-sEVs group were significantly higher than they were in the CTX + BUS group, and the FSH levels were lower than they were in the CTX + BUS group (*P* < 0.001). However, the levels of E_2_, FSH, and AMH in ESCs-sEVs group were not restored to the same level as those in the control group at 1 week (*P* < 0.05). After 2 weeks, the levels of E_2_, FSH, and AMH in the ESCs-sEVs group were restored to normal levels, which were the same as the control group (43.81 ± 2.43 mIU/mL vs 42.49 ± 2.39 mIU/mL; 15.62 ± 0.54 mIU/mL vs 12.88 ± 1.88 mIU/mL, and 1129.86 ± 40.21 pg/mL vs 1238.18 ± 54.64 pg/mL, respectively; *P* > 0.05). All the data suggested that ESCs-sEVs could promote the recovery of endocrine function in POF mice.

### Ovarian histological examination and follicle counts

**Fig. 3:**
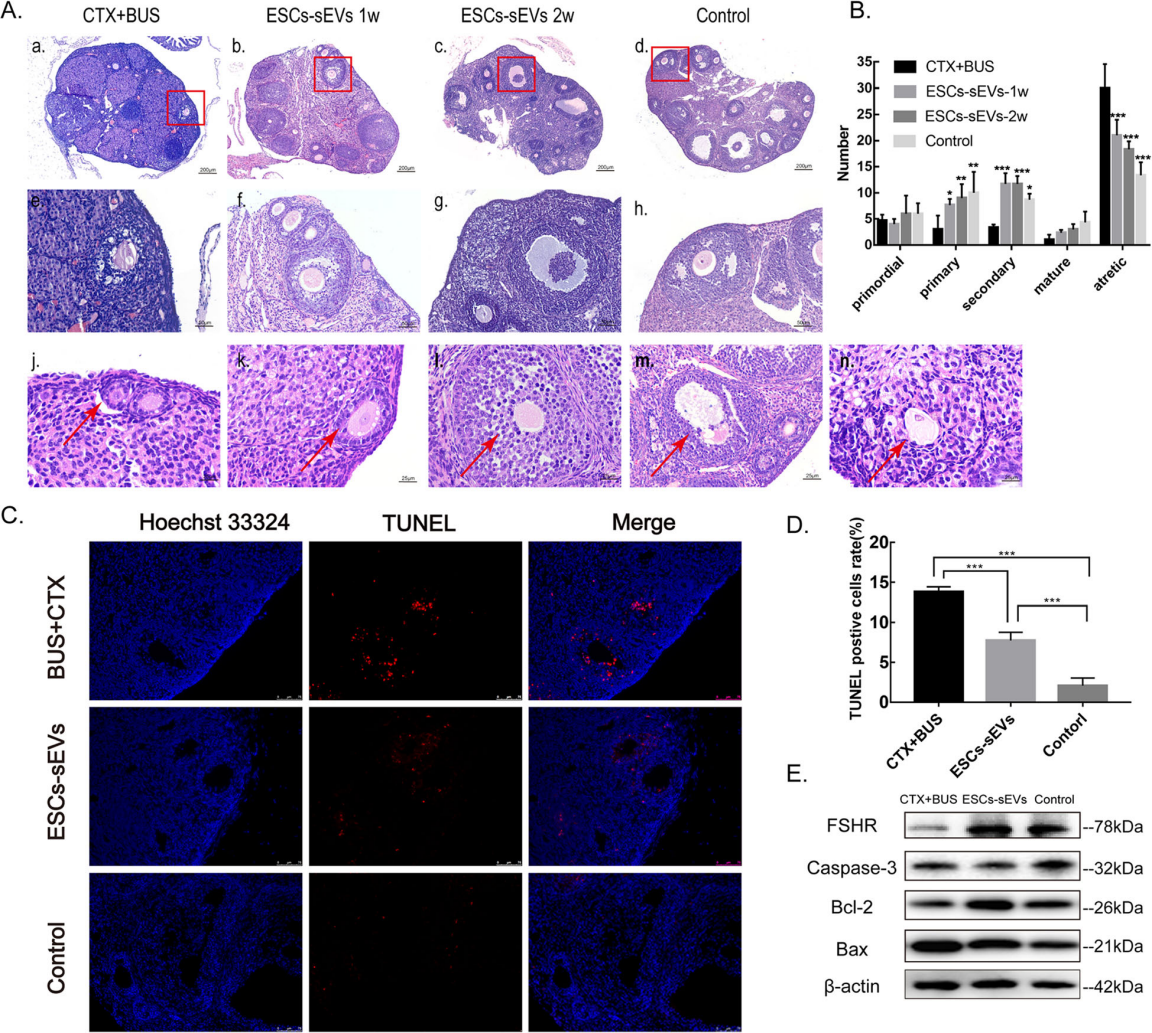
ESCs-sEVs promoted the development of follicles. **A** H&E staining of ovaries. **A** (e–h) Magnifications (× 100) of the red squares in **A** (a–d) (× 200), respectively. **A** (j) Primordial follicle. **A** (k) Primary follicle. **A** (l) Secondary follicle. **A** (m) Mature follicle. **A** (n) Atretic follicle. **B** Summary of follicles in each group. **C** Apoptosis was measured by TUNEL staining. The cell nuclei were stained by DAPI (blue fluorescence), and the apoptotic cells were stained with Cy3 (red fluorescence). **D** The percentage of TUNEL-positive granulosa cells was measured in each group. Apoptotic cells were increased in the CTX + BUS group and decreased in the ESCs-sEVs group. Scale bar = 75 μm **E** Western blotting analysis detected the expression of FSHR and a protein related to apoptosis in ovarian tissue. Data are presented as the mean ± SD. *P < 0.05, **P < 0.01, ***P < 0.001

To further observe histologic morphology, ovaries were collected at different time points, and the number of follicles was counted in a section (Fig. 3A). In the control group, follicles in all stages were observed: the primordial follicle (oocyte was surrounded by a layer of flat squamous granulosa cells) (Fig. 3A (j)), the primary follicle (oocyte was surrounded by one layer of cuboid granulosa cells) (Fig. 3A (k)), the secondary follicle (which was surrounded by more than one layer of cuboidal granulosa cells without visible antrum) (Fig. 3A (l)), the mature follicle (there is a large antral space, the cumulus is very obvious, and a radioactive crown appears) (Fig. 3A (m)), and the atretic follicle (the follicular wall collapses, the structure of the oocyte disappears, and the zona pellucida shrinks) (Fig. 3A (n)). Compared with the control group, the number of primordial cells declined without significance (4.67 ± 1.16 vs 6.00 ± 2.00, *P* > 0.05), but the primary and secondary follicles were significantly decreased (3.00 ± 2.65 vs 10.00 ± 4.00, *P* < 0.05; 3.33 ± 0.58 vs 8.67 ± 1.16, *P* < 0.01); further, the atretic follicles were significantly increased in the CTX + BUS group (30.00 ± 4.58 vs 13.33 ± 2.52, *P* < 0.001) (Fig. 3B). The ovarian interstitial tissue fibrosis was more severe, and the arrangement of granulosa cells in the follicles was irregular. After receiving the ESCs-sEVs treatment for 1 week, the primary and secondary follicles were significantly increased over what was observed in the CTX + BUS group (7.67 ± 1.16 vs 3.00 ± 2.64, *P* < 0.05; 11.67 ± 2.08 vs 3.33 ± 0.58, *P* < 0.001). The atretic follicles were significantly decreased (21.00 ± 3.00 vs 30.00 ± 4.58, *P* < 0.001), and the interstitial fibrosis was alleviated. After 2 weeks, the number of healthy follicles showed an increasing trend, especially the primary and secondary follicles, and the atretic follicles appeared to be in decline when compared to their levels at 1 week. In brief, our data indicated that transplantation of ESCs-sEVs might promote the development and survival of follicles and inhibit apoptosis, which contributed to the recovery of ovarian function.

### ESCs-sEVs suppressed the apoptosis of granulosa cells in the ovary

As shown in the H&E section, more atretic follicles could be observed in the CTX + BUS group than in the ESCs-sEVs group. To determine whether the CTX + BUS group had more apoptotic cells, in situ cell death detection was performed to analyze apoptosis in ovarian tissues. In the control group, few apoptotic cells were observed; a greater number of apoptotic cells were detected in the CTX + BUS group, and they were mostly located in the granulosa cell layer of follicles (13.83 ± 0.61% vs 2.10 ± 0.95%, *P* < 0.001) (Fig. 3C). Compared to the CTX + BUS group, the number of apoptotic cells were significantly reduced after treatment with ESCs-sEVs (7.76 ± 1.01% vs 13.83 ± 0.61%, *P* < 0.001) (Fig. 3D). Taken together, the follicular atresia in POF mice might be attributed to the apoptosis of granulosa cells. Furthermore, the ESCs-sEVs might suppress the apoptosis of granulosa cells caused by CTX + BUS, contributing to increased follicles. Next, the proteins related to apoptosis in mouse ovarian tissue were examined. In the CTX + BUS group, the results showed that the apoptotic proteins (Bax, Caspase-3) were increased and the anti-apoptotic protein (Bcl-2) and FSHR were decreased when compared to the control group. In the ESCs-sEVs group, the apoptotic proteins were decreased, and the anti-apoptotic protein and FSHR were significantly increased compared to the levels in the CTX + BUS group (Fig. 3E), indicating that the effect of ESCs-sEVs might be related to an anti-apoptotic effect.

### ESCs-sEVs restored ovarian function in a naturally aging mouse model

To further verify the establishment of the POF model, serum hormones were detected in aging mice (Additional file 2A). Two weeks after ESCs-sEVs treatment, the levels of E_2_ and AMH were significantly increased, and the FSH levels were significantly decreased (*P* < 0.001) compared to the aging-control group. Similar results were observed in the ovaries after H&E staining (Additional file 2B). Compared to younger ovaries, aging mice had smaller ovaries with rugged surfaces. In the aging + ESCs-sEVs group, the number of growth follicles was higher and the number of atretic follicles was lower than what was observed in the aging-control group. All the results demonstrated that the ESCs-sEVs play a similar role in both chemotherapy-induced POF mice and naturally aging mice.

### Morphology and identification of granulosa cells

**Fig. 4:**
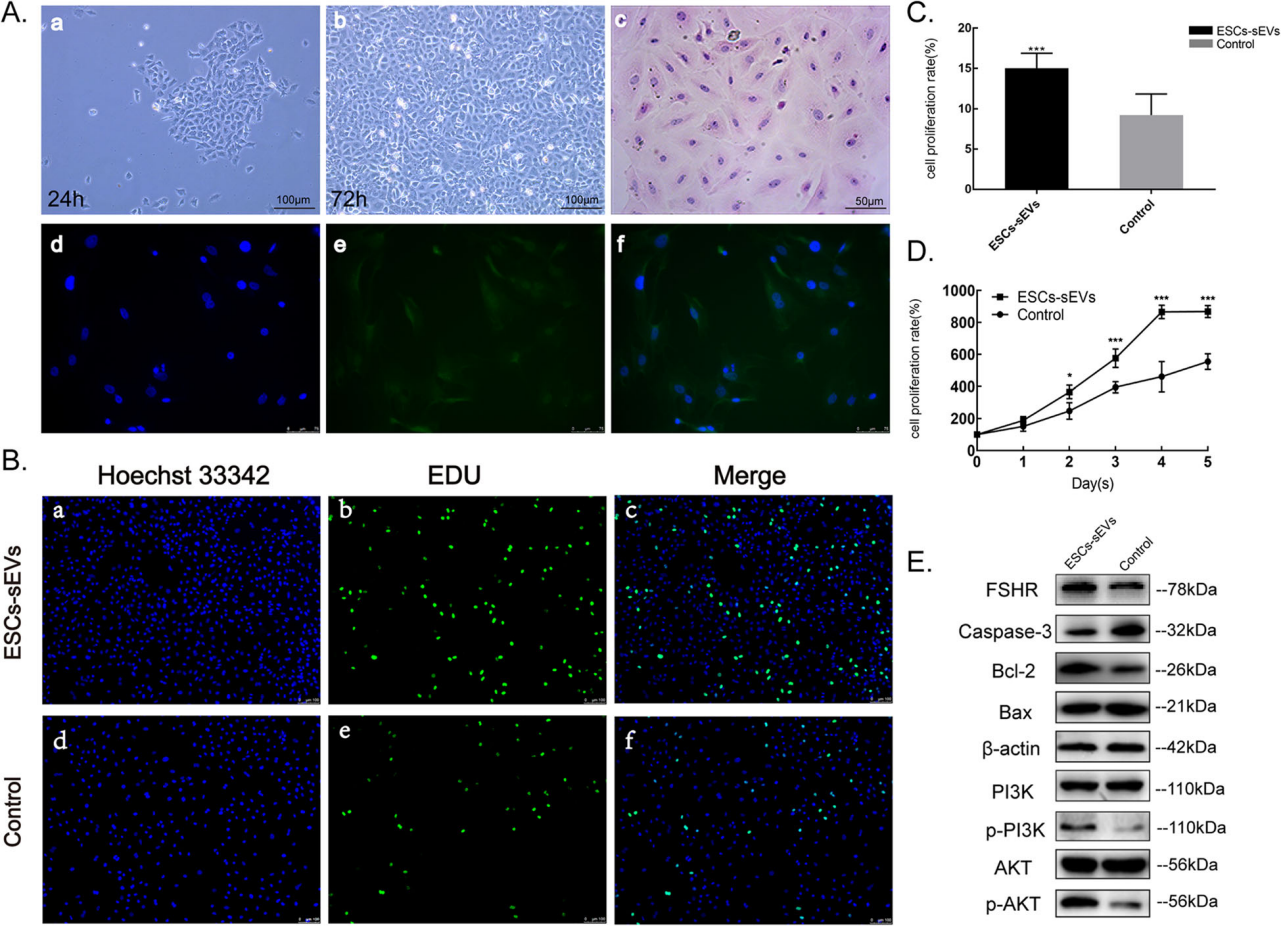
ESCs-sEVs promoted the proliferation and inhibited the apoptosis of ovary granulosa cells. **A** Morphology and identification of mouse ovarian granulosa cells. **A** (a) Granulosa cells adhered to the wall after 24 h (× 100). **A** (b) Granulosa cells proliferated significantly after 72 h (× 100). **A** (c) The phenotype of granulosa cells is shown by H&E staining (× 200), bars = 50 μm. **A** (d–f) Identification of ovarian granulosa cells by FSHR immunofluorescence (× 200). Bars = 100 μm. **B** ESCs-sEVs promoted the proliferation of granulosa cells, as detected by EdU tests; the cell nuclei are stained blue, and the cells with proliferation activity are stained green. **C** The proliferation ratio of granulosa cells was analyzed using EdU tests. **D** The cell proliferation curve of granulosa cells was measured by CCK-8 from day 1 to day 5. **E** The expression of FSHR, a protein related to apoptosis and the PI3K/AKT signaling pathway, was measured in granulosa cells by Western blotting analysis. Data are presented as the mean ± SD. *P < 0.05, **P < 0.01, ***P < 0.001

For the purpose of exploring possible mechanisms of ESCs-sEVs on POF, we extracted granulosa cells from mouse ovaries to culture in vitro. After 24 h of initial plating, granulosa cells showed a single layer of adherent growth (Fig. 4A (a)). The cells then showed significant proliferation after 72 h (Fig. 4A (b)). Under light microscopy analysis of H&E staining, the granulosa cells had oval or polygonal shapes and had blue nuclei and reddish cytoplasm (Fig. 4A (c)). FSHR is specifically expressed in the cytoplasm of granulosa cells, which can be used as a molecular marker to identify granulosa cells. Immunofluorescence staining suggested that nearly all the cells exhibited green fluorescence, which represents FSHR in the cytoplasm (Fig. 4A (d–f)). Hence, this characterization of the cells met the criteria for identifying them as of granulosa cells.

### ESCs-sEVs promoted the proliferation of granulosa cells in vitro

The proliferation of granulosa cells with/without ESCs-sEVs was measured by CCK-8 and EdU tests. In the EdU test (Fig. 4B), green fluorescence with FITC represented the proliferative activity of granulosa cells, and the proliferation rate was significantly increased in the ESCs-sEVs group compared to the control group (14.94 ± 1.86% vs 9.21 ± 2.62%, *P* < 0.001) (Fig. 4C). Similar results were observed using the CCK-8 test (Fig. 4D). The proliferation of granulosa cells was significantly enhanced by stimulating with ESCs-sEVs for 5 days, especially from days 2 to 4. The abovementioned results indicated that the ESCs-sEVs could promote the proliferation of granulosa cells in vitro.

### ESCs-sEVs promoted the PI3K/AKT pathway and inhibited cell apoptosis of granulosa cells

The PI3K/AKT signaling pathway has been reported to play a critical role in the survival and activation of primordial follicles that affect folliculogenesis. To determine whether PI3K/AKT was involved in the ovarian repair in POF mice, proteins related to the PI3K/AKT signaling pathway and apoptosis were detected by Western blotting in granulosa cells. As shown in Fig. 4E, phosphorylated PI3K and AKT were increased in the ESCs-sEVs group when compared to the control group, indicating the activation of the PI3K/AKT signaling pathway. In addition, the expression of Bax and Caspase-3 was decreased, and Bcl-2 was increased in granulosa cells compared to the control group, which was consistent with the observations from the ovarian tissue. Meanwhile, higher expression of FSHR located on the cell membrane surface of granulosa cells could be observed in both ovarian tissues and granulosa cells.

### The effect of ESCs-sEVs on granulosa cells was inhibited by a PI3K/AKT inhibitor

LY294002 was used as an inhibitor of AKT phosphorylation in cultured granulosa cells with/without ESCs-sEVs to investigate whether the PI3K/AKT signaling pathway influenced the proliferation, apoptosis, and secretion function of granulosa cells in vitro.

### Effect of LY294002 on the proliferation of granulosa cells with/without ESCs-sEVs

**Fig. 5:**
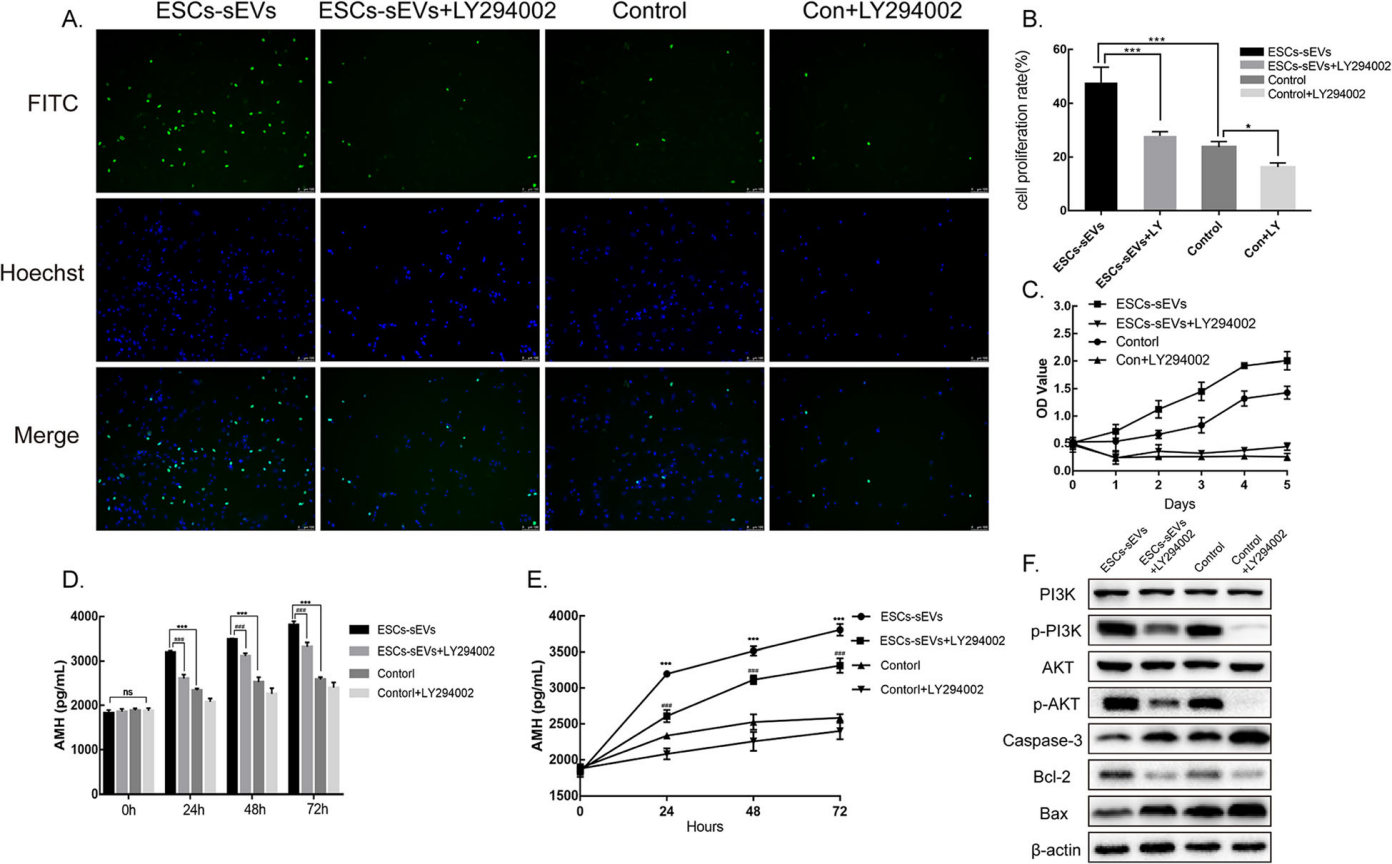
The effect of ESCs-sEVs on granulosa cells was inhibited by a PI3K/AKT inhibitor. **a** ESCs-sEVs promoted the proliferation of granulosa cells, while the PI3K/AKT pathway inhibitor LY294002 reversed this effect. **b** The proliferation ratio of granulosa cells with/without ESCs-sEVs or LY294002. **c** CCK-8 tests indicated that the proliferation activity of granulosa cells was obviously inhibited in the presence of LY294002 as the time passed, which was consistent with the EdU test. **d** ESCs-sEVs increased the level of AMH synthesized by granulosa cells, while it was suppressed by LY294002. **e** The AMH secretion curve is shown in different groups at 24 h, 48 h, and 72 h. **f** ESCs-sEVs increased the phosphorylation of PI3K and AKT, as detected by Western blotting, and they inhibited the downstream apoptosis pathway. The results are presented as the mean ± SD. *P < 0.05, **P < 0.01, ***P < 0.001, ESCs-sEVs versus control group. ^###^*P* < 0.001, ESCs-sEVs versus ESCs-sEVs + LY294002 group

The proliferation of granulosa cells in different conditions was evaluated by EdU and CCK-8 tests. The EdU results showed (Fig. 5a, b) that the effect that ESCs-sEVs had on enhancing proliferation activity nearly disappeared after treatment with LY294002 (47.81 ± 5.55% vs 27.94 ± 1.60%, ESCs-sEVs vs ESCs-sEVs + LY294002 group, *P* < 0.001). Similar results were observed by CCK-8 tests (Fig. 5c). All these data suggested that the effect of promoting proliferation on granulosa cells was suppressed when the PI3K/AKT pathway was blocked.

### Effect of LY294002 on the AMH supernatant levels for granulosa cells with/without ESCs-sEVs

The supernatant of granulosa cell culture medium with/without ESCs-sEVs or LY294002 was analyzed by ELISA. Compared to the ESCs-sEVs group, the concentration of AMH was significantly decreased in both the ESCs-sEVs + LY294002 and control + LY294002 groups (Additional file 3), which meant that ESCs-sEVs promoted the secretion from granulosa cells, and it was significantly inhibited by treatment with the PI3K/AKT pathway inhibitor (Fig. 5d, e).

### Effect of LY294002 on the PI3K/AKT signaling pathway and protein related to cell apoptosis in granulosa cells under different conditions

As shown in Fig. 5f, phosphorylation of PI3K and AKT was increased in the ESCs-sEVs group, and the expression of downstream apoptotic proteins was decreased, while the anti-apoptotic protein was increased. However, the inverse results were obtained when cells were in the presence of a signaling inhibitor (both the ESCs-sEVs + LY294002 and control + LY294002 groups). These findings showed that ESCs-sEVs affect granulosa cells by promoting the PI3K/AKT pathway.

## Discussion

For the past few years, various stem cell treatments for POF have been reported, however, few studies on EVs derived from stem cells for the treatment of POF have been performed. In our experiment, the results suggested that ESCs-sEVs restored hormones E_2_, AMH, and FSH to normal levels; decreased the atretic follicles; and increased the number of normal follicles. Further studies have found that ESCs-sEVs could promote the PI3K/AKT pathway and increase PI3K and AKT phosphorylation, thereby inhibiting apoptosis and promoting proliferation of granulosa cells.

POF is defined as cessation of ovarian function due to early amenorrhea, i.e., before 40 years old, and not due to pregnancy, and it involves increased FSH and decreased E_2_ levels [16]. Among women younger than 40 years, the incidence rate is approximately 1/100, while it is 1/1000 for those under 30 years and 1/10000 for those under 20 years. In recent years, the incidence rate has increased gradually and tended to be in younger patients [1]. The etiology of POF remains unknown until now and is mostly related to genetics, autoimmunity, chemoradiotherapy, and environmental factors. Chemotherapy is commonly used in patients with cancers that contributed mostly to POF [17].

POF caused by chemotherapy and radiotherapy has gained wide attention in recent years. Chemotherapy kills the tumor cells by inducing programmed cell death or apoptosis. It also causes apoptosis in other normal tissues, and these mainly involve the ovaries; cytotoxic drugs, such as cyclophosphamide, cause the greatest damage to the ovaries [18]. Cyclophosphamide excessively activates the dormant primordial follicles in the ovary, accelerating the growth of atretic follicles. This eventually leads to depletion of the ovarian reserve, causing POF [19]. Similarly, busulfan could also cause damage to ovarian function due to its cytotoxic effects [20]. Studies have shown that inflammation and ischemia can be observed in heart, liver, kidney, and spleen tissues of mice after receiving chemotherapy for a week, while changes in each organ could be relieved after 2 weeks without any treatment. However, the damage to ovarian tissue was persistent and irreversible without undergoing any therapy, and the results involve ovulation disorders, tissue fibrosis, and even infertility [21]. In patients with POF, follicles showed dysplasia, and the atretic follicles were increased; additionally, the follicles could be atretic when over 10% of granulosa cells showed apoptosis [22]. This indicated that apoptosis of granulosa cells is the main mechanism of follicular atresia at various stages of development [23]. Furthermore, granulosa cells provide necessary growth signals and nutrients for oocyte maturation. The state of the granulosa cells is important for follicular development, which essentially reduces apoptosis of granulosa cells and repair of the primordial follicle reserve pool and is the main mechanism of repairing ovarian function. Therefore, investigating the effect of ESCs-sEVs in granulosa cells assists in clarifying the mechanism of ESCs-sEVs in treating POF. In our study, increased apoptosis in granulosa cells and atretic follicles were observed in POF animal models, while apoptotic cells and atretic follicles were significantly reduced and growth follicles were significantly increased after treatment with ESCs-sEVs, especially the primary and secondary follicles, suggesting that ESCs-sEVs could inhibit the apoptosis of granulosa cells and atresia of follicles. In addition, similar experiments were conducted in naturally aging mice to verify the validity of the animal model treated with chemotherapy, and promising results were observed.

ESCs-sEVs have been reported to play an important role in accelerating wound healing by reducing endothelial senescence [11], but the function of EVs in the ovary has not been investigated. The PI3K/AKT pathway contributes to metabolism, cell homeostasis, neurodevelopment, and other processes, and it regulates various aspects of cell development, such as apoptosis, cell cycle, and cell differentiation. In addition, the PI3K/AKT pathway participates in oocyte growth, primordial follicular development, and granulosa cell proliferation and apoptosis. In previous studies, cytotoxic drugs were found to lead to apoptosis of granulosa cells and increase the risk of developing POF by significantly inhibiting the PI3K/AKT signaling pathway [24]. However, whether PI3K/AKT signaling is involved in ESCs-sEVs treatment for POF remained to be further explored. Therefore, this study was conducted by culturing ovarian granulosa cells with PI3K/AKT signaling pathway inhibitors to observe the effect of ESCs-sEVs during the process of proliferation and apoptosis of granulosa cells. The cell proliferation experiments demonstrated that the proliferation of granulosa cells was significantly enhanced with ESCs-sEVs and weakened by treatment with ESCs-sEVs and signal inhibitors. Moreover, we also examined the expression of apoptosis-related proteins, including Bax, Bcl-2, and caspase-3. Bax and Bcl-2 belong to the Bcl-2 family, which play a critical part in the mitochondrial apoptosis pathway, and they have been shown to regulate apoptosis in the ovary [25]. Bax protein is located in the cytoplasm and can translocate to the mitochondrial membrane, forming mitochondrial porin channels and promoting the release of cytochrome C (cyt-C), resulting in the apoptosis of cells. Bcl-2 was found to exert an anti-apoptotic effect by forming heterodimers with Bax. It has been reported that the overexpression of Bcl-2 could antagonize the effect of Bax and inhibit the apoptosis of ovarian granulosa cells [26]. The caspase family also plays an important role in the process of apoptosis. Caspase-3 is a key apoptotic executive enzyme downstream of the caspase cascade. Its activation is largely dependent on the release of cyt-C, resulting in the apoptosis of various cells [27]. In our study, the expression of proapoptotic proteins (Bax and caspase-3) was significantly reduced, while the expression of an anti-apoptotic protein (Bcl-2) was significantly increased following treatment with ESCs-sEVs. Further analysis of proteins related to the PI3K/AKT pathway revealed that ESCs-sEVs increased the phosphorylation of AKT, a molecule downstream of PI3K, which meant that the PI3K/AKT pathway was activated. These findings are consistent with a previous study regarding the repair of ovarian function by MSCs through PI3K/AKT pathway activation [28]. Therefore, we speculated that the PI3K/AKT pathway was downregulated under the influence of CTX and BUS, promoting Bax translocation to mitochondria, promoting the release of cytochrome C, and triggering apoptosis [29]. Hence, ESCs-sEVs might prevent this effect by activating the PI3K/AKT pathway. However, it is still unclear how ESCs-sEVs activate the PI3K/AKT pathway, and this problem requires further investigation in our next study.

## Conclusions

In summary, our present findings demonstrated that ESCs-sEVs could recover ovarian function in chemotherapy-induced POF and inhibit apoptosis of granulosa cells by activating the PI3K/AKT pathway. Moreover, our study could provide a promising therapeutic approach for POF in the clinic.

### Supplementary information

**Additional file 1.** The hormones of different groups of mice. Levels of E_2_, FSH and AMH were measured by ELISA at 0 week (the day before treatment), 1 week and 2 weeks.

**Additional file 2. **ESCs-sEVs restored ovarian function in a naturally aging mouse model. A-a E_2_ was significantly increased compared to the Aging-control group. A-b FSH was significantly decreased compared to the Aging-control group. A-c AMH was significantly increased compared to the Aging-control group. B H&E staining for ovaries. B-a Aging mice after ESCs-sEVs treatment. B-b Aging mice without ESCs-sEVs treatment. B-c Eight-week old normal mice. Scale bar = 300 μm. ^***^P < 0.001, control versus ESCs-sEVs group.

**Additional file 3.** The level of AMH secreted by granulosa cells at different points.

## Data Availability

The datasets used and/or analyzed during the current study are available from the corresponding author on reasonable request.

## Abbreviations

AMH: Anti-Mullerian hormone
BUS: Busulfan
CTX: Cyclophosphamide
E_2_: Estradiol
ESCs-sEVs: Small extracellular vesicles derived from embryonic stem cells
FBS: Fetal bovine serum
FSH: Follicle stimulating hormone
H&E: Hematoxylin and eosin
HRT: Hormone replacement therapy
PBS: Phosphate-buffered saline
POF: Premature ovarian failure
TEM: Transmission electron microscope
