# Correction: Small extracellular vesicles derived from embryonic stem cells restore ovarian function of premature ovarian failure through PI3K/AKT signaling pathway

**DOI:** 10.1186/s13287-023-03512-3

**Published:** 2023-09-29

**Authors:** Mengyu Liu, Yu Qiu, Zhuowei Xue, Ruoyu Wu, Jie Li, Xin Niu, Ji Yuan, Yang Wang, Qingkai Wu

**Affiliations:** 1 Department of Obstetrics and Gynecology, Shanghai Jiao Tong University Affiliated Sixth People’s Hospital, No.600 Yishan Road, Shanghai, 200233 China; 2 Medical College of Soochow University, Suzhou, 215006 China; 3 Institute of Microsurgery On Extremities, Shanghai Jiao Tong University Affiliated Sixth People’s Hospital, No.600 Yishan Road, Shanghai, 200233 China

**Correction : Stem Cell Research & Therapy (2020) 11:3** 10.1186/s13287-019-1508-2

Following the publication of the original article [1], the authors have identified that the Actin blot in Fig. 1C was duplicated from the GM130 blot due to an error during figure preparation. The correct Actin blot has been provided in Fig. 1C, and the correction does not change the conclusion of the article. The authors apologize for any inconvenience caused.

**Fig. 1:**
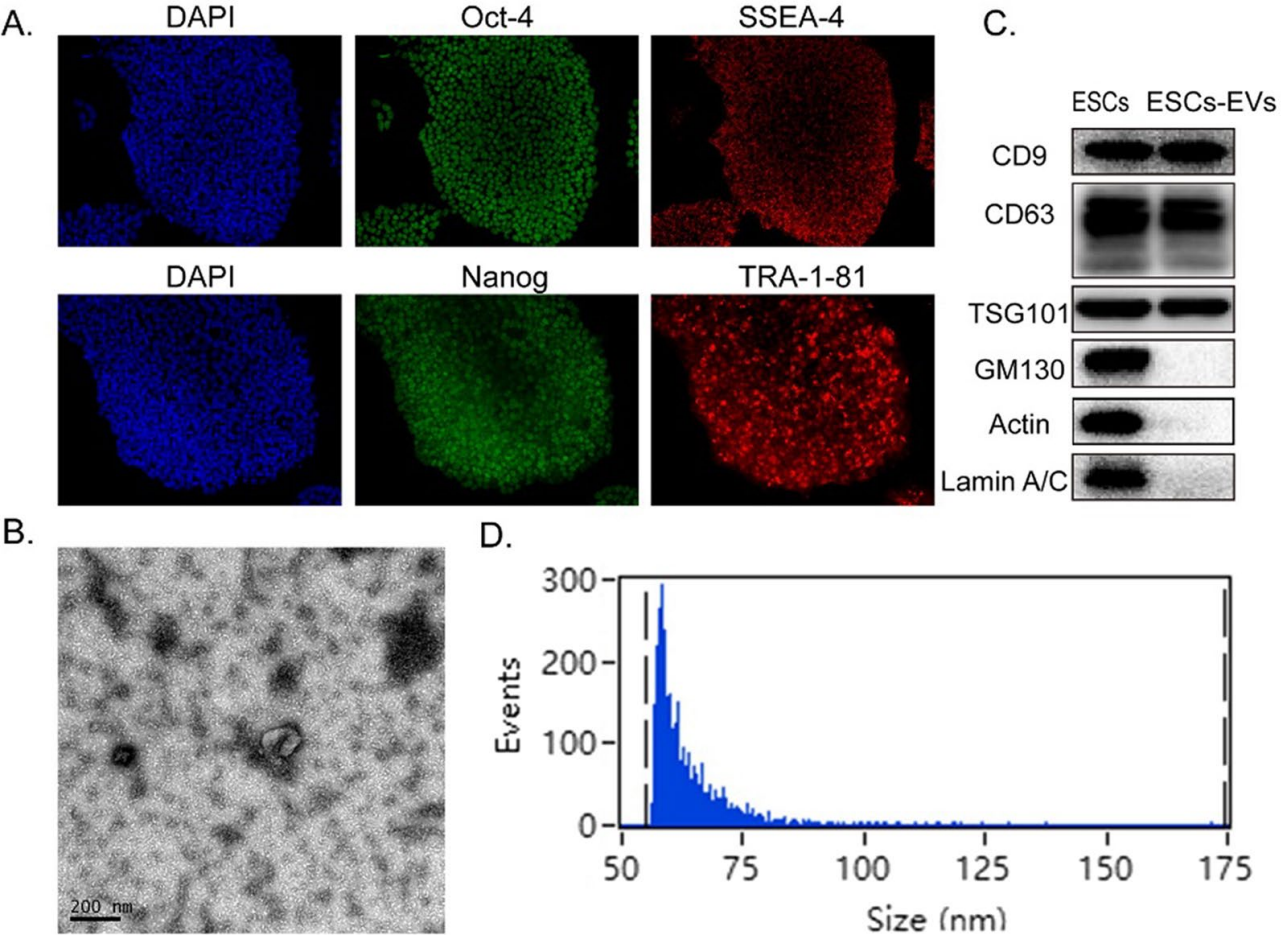
Characterization of ESCs and ESCs-sEVs. **a** Immunofluorescence detected the pluripotency markers in ESCs, including Oct-4, SSEA-4, Nanog, and TRA-1–81. Scale bars = 50 μm. **b** The morphology of ESCs-sEVs by TEM. Scale bars = 200 nm. **c** ESCs-sEVs were positive for CD9, CD63, and TSG101 and negative for GM130, Actin, and Lamin A/C, as shown by Western-blotting analysis. **d** Particle size distribution of ESCs-sEVs was determined by Flow Nano Analyzer

In addition, the animal experiments were approved by Animal Welfare Ethics Committee of Shanghai Sixth People's Hospital, the approval number was No: 2018–0060.
