# Supplementary material for: Small extracellular vesicles derived from embryonic stem cells restore ovarian function of premature ovarian failure through PI3K/AKT signaling pathway
Source: Stem Cell Res Ther. 2020 Jan 3;11:3. doi: 10.1186/s13287-019-1508-2 (PMC6942273; doi:10.1186/s13287-019-1508-2)
Supplement: Supplementary file 2 — Additional file 2. ESCs-sEVs restored ovarian function in a naturally aging mouse model. A-a E2 was significantly increased compared to the Aging-control group. A-b FSH was significantly decreased compared to the Aging-control group. A-c AMH was significantly increased compared to the Aging-control group. B H&E staining for ovaries. B-a Aging mice after ESCs-sEVs treatment. B-b Aging mice without ESCs-sEVs treatment. B-c Eight-week old normal mice. Scale bar = 300 μm. ***P < 0.001, control versus ESCs-sEVs group. [file 13287_2019_1508_MOESM2_ESM.docx]

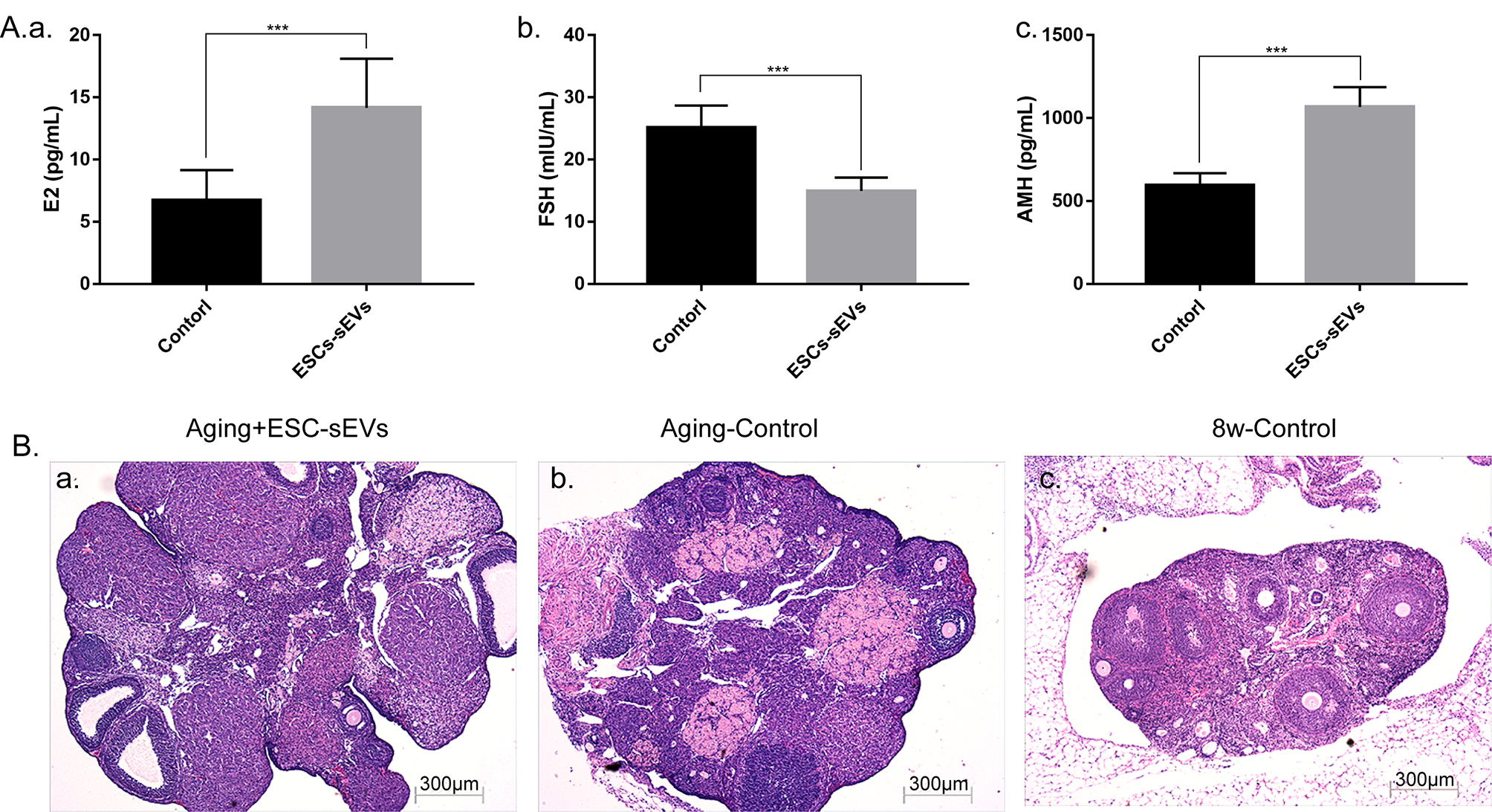
**Additional file 2.** ESCs-sEVs restored ovarian function in naturally aging mice model. **A-a** E2 was significantly increased compared to Aging-control group. **A-b** FSH was significantly decreased compared to Aging-control group. **A-c** AMH was significantly increased compared to Aging-control group. **B** H&E staining for ovaries. **B-a** Aging mice after ESCs-sEVs treatment. **B-b** Aging mice without ESCs-sEVs treatment. **B-c** Eight-week normal mice. Scale bar=300μm.^***^*P*<0.001,control versus ESC-sEVs group.
